# Advances in Stem Cell Research- A Ray of Hope in Better Diagnosis and Prognosis in Neurodegenerative Diseases

**DOI:** 10.3389/fmolb.2016.00072

**Published:** 2016-11-08

**Authors:** Shripriya Singh, Akriti Srivastava, Pranay Srivastava, Yogesh K. Dhuriya, Ankita Pandey, Dipak Kumar, Chetan S. Rajpurohit

**Affiliations:** ^1^System Toxicology and Health Risk Assessment Group, Council of Scientific and Industrial Research-Indian Institute of Toxicology ResearchLucknow, India; ^2^Academy of Scientific and Innovative ResearchLucknow, India

**Keywords:** neurodegenerative disorders, stem cells, Induced pluripotent stem cells (iPSCs), disease modeling, diagnosis

## Abstract

Neurodegeneration and neurodegenerative disorders have been a global health issue affecting the aging population worldwide. Recent advances in stem cell biology have changed the current face of neurodegenerative disease modeling, diagnosis, and transplantation therapeutics. Stem cells also serve the purpose of a simple *in-vitro* tool for screening therapeutic drugs and chemicals. We present the application of stem cells and induced pluripotent stem cells (iPSCs) in the field of neurodegeneration and address the issues of diagnosis, modeling, and therapeutic transplantation strategies for the most prevalent neurodegenerative disorders. We have discussed the progress made in the last decade and have largely focused on the various applications of stem cells in the neurodegenerative research arena.

## Introduction

Progress in the field of clinical research and medicine has decreased global mortality drastically. The developed countries have extended the life span of their aging population. However, the modern world is now faced with the issues of aging and age related disorders. Neurodegeneration and neurodegenerative disorders are one of the major health implications faced by the aging population. Neurodegeneration studies have largely benefited from neuropathology and *in-vivo* research (Agrawal and Biswas, 2015). Neurodegenerative disorders have been thoroughly investigated using animal models, primary cultures, and post mortem human brain tissues (Marchetto et al., 2011). Though informative, these approaches have some limitations. Data obtained from animal models fails to directly correlate with that of humans because a rodent brain is not an exact mimic of a human brain. Despite being highly conserved evolutionarily, mammalian genomes are not identical. The embryonic development of mice and humans are considerably different and almost 20% genetic variability is accounted for (Strachan et al., 1997). Therefore species' difference prevents the animal data from successful validation during clinical field trials which poses a severe economic burden. A study reported the failure of therapeutic drugs for treating amyotrophic lateral sclerosis in human beings, which had earlier proved effective in case of rodents (Takahashi and Yamanaka, 2013). Preclinical studies often do not efficiently translate to the clinic and the clinical trial failures have been reported time and again (Prinz et al., 2011; Begley and Ellis, 2012). Primary culture of neurons is challenging because these are the post mitotic differentiated cells which are difficult to sustain in the *in-vitro* conditions. Ethical constraints have held back human based research and thus the best possible source of human samples are the postmortem brain tissues. However, these autopsied samples depict the end stages of the disease and do not give much insight into the intricacies of the disease' developing stages (Marchetto et al., 2011). Researchers are not willing to subject the human beings to untested interventions, but the choices have been limited so far.

Majority of neurodegenerative disorders have been incurable (Alzheimer's disease, Parkinson's disease, Huntington's disease, Amyotrophic lateral sclerosis) so far but timely diagnosis can help in the management and symptom alleviation. However, researchers across the world are continuously striving to achieve the cure and hope to achieve fruitful results in the near future. Neurodegeneration studies are largely divided into two major categories. One is the experimental modeling strategy which allows for a comprehensive understanding of the disease such as the etiology, pathophysiology, genotypic-phenotypic interactions, symptomatic, and mechanistic insights. The second is the medical approach which deals with the treatment, therapy, and disease management. Stem cells and iPSCs find widespread application for both, disease modeling as well as transplantation and regenerative therapeutics. In the present review we shall discuss the applicability of stem cell research in the field of neurodegenerative disease modeling and provide the current updates of how stem cell and induced pluripotent stem cell based studies have been employed to address the diagnosis and therapy of the most common neurodegenerative disorders. We shall briefly touch upon the advances and preferable methodologies employing stem cell and iPSC culture such as the three dimensional (3D) culture which has revolutionized the current trend of *in-vitro* studies. The article intends to highlight the fact, that though animal based *in-vivo* research is absolutely necessary for the neuroscience research, one cannot wholly and solely depend upon it and human based stem cell driven research has and will open newer avenues for the neurodegenerative disorders′ modeling and treatment.

## Stem cells and induced pluripotent stem cells (IPSCs) in neurodegeneration: why the choice?

It is easier to say that cells of human origin can be directly employed to generate a clearer picture of the neurodegenerative diseases but practically the approach is not as simple as it seems. The *in-vitro* scenario is devoid of an intact organ system, organ-organ interactions are missing and the blood supply and connective tissues are lacking. Every disease has its characteristic cellular, molecular, anatomical, genotypic, and phenotypic attributes. If one has to model these various aspects *in-vitro*, very specific cell types expressing the disease phenotypes are required. Sustaining the culture of such specific cells is another challenging task that requires standardized protocols, select growth conditions, and expertise. For example, Parkinson's disease requires the culture of dopaminergic neurons, ALS requires the culture of glial cells, motor neurons, and astrocytes and Huntington's disease requires the culture of medium spiny and striatal projection neurons as discussed in the later sections. All these requirements have been largely met by the use of stem cell technology.

Stem cells in brief are the naïve cells of the body with an exceptional ability to self-renew, proliferate, differentiate, and get programmed for multi-lineage commitment (Cananzi and De Coppi, 2012; Liu et al., 2013; Kumar et al., 2015). Their origin can either be fetal, embryonic, or adult tissues of the body (Nam et al., 2015; Singh et al., 2015). Despite a few ethical concerns stem cell biology is finding widespread applicability in the field of research and medicine. Stem cells can be practically converted into any possible cell type and thus are being routinely used to model diseases. Monogenic disorders with a clear cut cellular phenotype and high penetrance are comparatively easier to model than the late onset diseases involving a number of genes and showing less penetrance. In case of the monogenic disorders the disease associated gene is deliberately mutated via gene editing to obtain the stem cell models. Embryonic stem cells (ESCs) harboring the chromosomal aberrations are used for the modeling of the chromosomal diseases. The late onset complex diseases which cannot be prenatally diagnosed are modeled using patient derived iPSCs more effectively (Avior et al., 2016).

*In-vivo* animal models have so far been used to experimentally model diseases however, the data generated fails to recapitulate the human diseases and thus cannot be directly extrapolated (Yamanaka, 2009). This forms a major limitation of the various animal based studies. Only samples of human origin can be employed to overcome this major hurdle. Neurodegeneration leads to a gradual loss of brain functionality via an irreversible gradual loss of neurons and other cells of the central nervous system (Peng and Zeng, 2011). In this regard transplantation therapy is employed to restore and repair the damaged circuitry of the brain as well as to replenish the lost neuronal population (Thompson and Björklund, 2015). Successful commitment of stem cells toward the neuronal lineage is widely reported and myriad of protocols are available to achieve the same (Nikoletopoulou and Tavernarakis, 2012; Ferroni et al., 2013; Lu et al., 2015).

**Figure 1:**
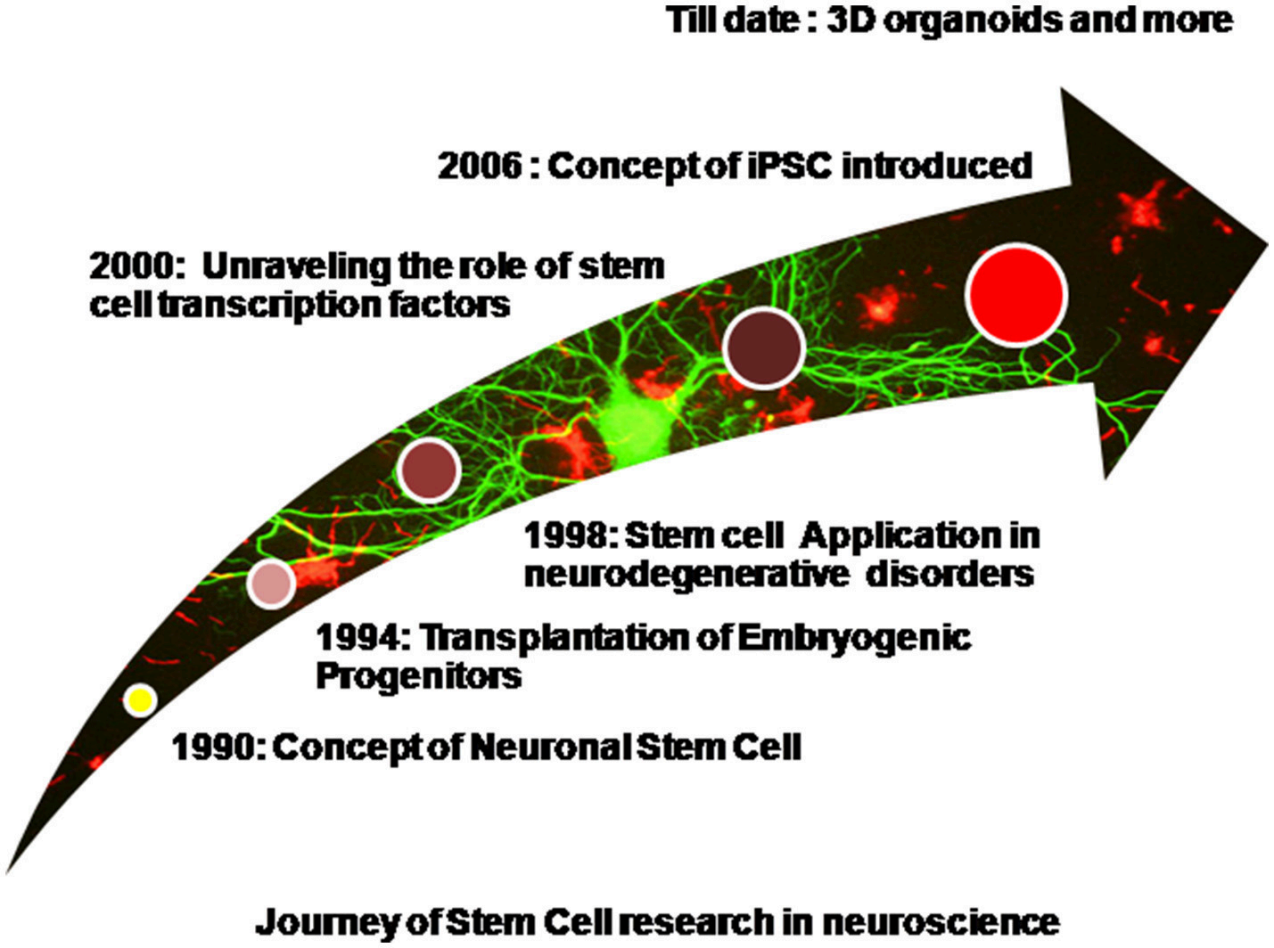
**Diagrammatic representation of progress made in the field of stem cell research in context with neuroscience**.

Diseases such as brain ischemia (Ju et al., 2014), spinal muscular atrophy (Frattini et al., 2015), spinal cord injury (Lukovic et al., 2015), amyotrophic lateral sclerosis (Nicaise et al., 2015), Machado-Joseph disease (Mendonça et al., 2015), and many more have been studied and stem cell therapy has been effectively employed for the same. Embryonic stem cells are pluripotent in nature and hold an excellent potential for restoring brain injuries and neurodegeneration via transplantation therapy, however tumor formation restricts their widespread application (Aleynik et al., 2014). Mesenchymal stem cells which are multipotent also find widespread application because they are immunomodulating in nature. Immunomodulation simply refers to the unique ability to escape the host's immune system surveillance thereby leading to successful transplants without eliciting an adverse immune response (Glenn and Whartenby, 2014). Neural progenitor cells or NSCs isolated from fetal brain are again multipotent in nature and are stringently committed toward the neuronal lineage. They are suitable because of a reduced risk of tumor formation however they are difficult to procure and usually few in number (Jiang et al., 2012). Apart from naïve stem cells, iPSCs derived via a reverse programming of somatic cells finds widespread application now a days (Takahashi and Yamanaka, 2006). Induced pluripotent stem cells are being produced in bulk and various iPSC cell lines are commercially made available. The use of Parp1 i.e., poly(ADP-ribose) polymerase 1 for the production of iPSCs is now well reported and this has also reduced the tumor forming risk sufficiently (Chiou et al., 2013). However, the formation of teratomas has not been completely eliminated. Production of iPSCs is well reported from various somatic cells of the body such as the peripheral blood cells, hepatocytes, stomach cells (Okano et al., 2013), and keratinocytes (Aasen et al., 2008). It is now well reported that just 10 μl of capillary blood drawn from finger tips can be used to generate iPSCs (Tan et al., 2014). Patient derived induced pluripotent stem cells are widely used cells of human origin which can directly be used to model the various human neurodegenerative diseases (Sterneckert et al., 2014). Scientific groups have reported disease specific iPSCs cell lines (Dimos et al., 2008). Stem cells and induced pluripotent stem cells have been widely used for modeling several neurodegenerative diseases as well as used in transplantation therapy. Spinal muscular atrophy has been efficiently modeled *in-vitro* using patient derived iPSCs (Sareen et al., 2012; Wang et al., 2013). The first of their kind, these iPSCs derived models efficiently depicted the disease phenotypes. However, iPSCs too have a few limitations. Neurodegenerative disorders are generally the late-onset diseases and symptoms begin to manifest with increasing age. Thus modeling such diseases via the animal models is not only time consuming but also cost heavy. It is generally assumed and even reported that patient derived iPSCs harbor the disease mutations and carry the epigenetic background of the patient, thus making them an excellent choice as the *in–vitro* disease models. However, when somatic cells are reprogrammed to an induced pluripotent state they lose the age associated features, undergo rejuvenation, and their embryonic age is reestablished. It is well reported that even aged donor derived iPSCs are rejuvenated and age reversal is evident as there is a loss/decrease in the markers of senescence, enhanced mitochondrial fitness, and an increased telomere length (Lapasset et al., 2011; Freije and López-Otín, 2012). Thus even these patient derived iPSCs do not effectively model the late onset neurodegenerative diseases as they lack the age related phenotypes. However, this hurdle has been largely overcome by progerin induced aging in the iPSCs (Miller et al., 2013). Progerin is a truncated transcript of lamin A (nuclear envelop protein) formed by mutation in the gene LMNA. Accumulation of progerin in the nuclear membrane results in the dysfunction of lamin A resulting in interrupted chromatin organization, cell cycle, telomere maintenance, and DNA damage response. High progerin levels are associated with aging. Age related phenotypes have been observed in the progerin exposed iPSCs such as degeneration of dendrites, neuromelanin accumulation, AKT deregulation, mitochondrial swelling, and reduction in the TH-positive neurons (Miller et al., 2013). These iPSCs harboring the aging markers will thereby mimic the neurodegenerative disorders more efficiently. In the upcoming sections we shall discuss the application of stem cells and iPSCs for the most common and globally prevalent neurodegenerative disorders. Figure 1 shows a diagrammatic representation of progress made in the field of stem cell research in context with neuroscience.

## Alzheimer's disease (AD)

Alzheimer's disease (AD) is described as “Presenile dementia” by German psychiatrist Alois Alzheimer and is one of the most prevalent neurodegenerative disorders of the world. It is a leading cause of dementia in the aging population and has lately been declared the sixth major reason for death. Patients having AD are reflected with cognitive deficits, memory loss, and behavioral changes and these changes are inherently associated with neurodegeneration (Blundell and Shah, 2015). Hippocampus, amygdala, neocortex, and basal forebrain regions of the brain are adversely affected leading to the severe impairment of cognition and memory. Neurofibrillary tangles (NFT) and β-amyloid plaques are the pathological hallmarks of AD (Peng and Zeng, 2011). Hyperphosphorylation of tau proteins and amyloid peptide aggregates are responsible for the formation of NFT and β-amyloid plaques respectively. Alzheimer genetics involves the mutated forms of presenilin 1 (PSEN1), presenilin 2 (PSEN2), Amyloid Precursor Protein (APP), and apolipoprotein E. There are still no permanent treatments available for AD except acetylcholinesterase (AChE) inhibitors which provide only temporary relief (Birks, 2006; Lindvall and Kokaia, 2006). Several drugs serve as potent acetylcholinesterase inhibitors such as tacrine, tacrine derivatives, donepizel, rivastigmine, galantamine, and the glutamate receptor agonist memantine (Romero et al., 2013; Cecilia Rodrigues Simoes et al., 2014; Ehret and Chamberlin, 2015). These FDA approved pharmacological interventions provide only symptomatic relief for a limited period and may also have side effects in the long run. Removal of the Amyloid β levels from the brain is considered an effective therapy for AD and physiologically, the enzyme neprilysin has been reported to be involved in the clearance of the Amyloid β plaques by degrading it (Iwata et al., 2001). Other proteinases such as cathepsin B (Mueller-Steiner et al., 2006) and plasmin (Melchor et al., 2003) too have a similar role and are used to decrease the levels of Aβ thus acting as potent therapeutic agents for AD. A number of past studies show the relevance of nerve growth factor (NGF) in the prevention of neurodegeneration and amyloid toxicity (Tuszynski et al., 2005; Tuszynski, 2007) but a severe limitation with NGF is that it is unable to cross the blood brain barrier and therefore cannot be delivered peripherally.

Transgenic animal models of AD carrying the disease mutations have given ample insight into the etiology and pathophysiology of the disease but have failed to entirely recapitulate the formation of NFT and β-amyloid plaques together. Human disease pathophysiology has not been completely depicted by mouse models so far (D'avanzo et al., 2015; Pistollato et al., 2015) and clinical failure of drugs has also been reported (Cavanaugh et al., 2014; Langley, 2014). Past studies demonstrate that the implantation of genetically modified fibroblast cells into the forebrain of the patients led to decreased neurodegeneration and restored the cognitive deficits associated with AD (Tuszynski et al., 1994). However, fibroblasts are immobile in nature and cannot migrate efficiently within the different brain regions, but on the other hand transplanted stem cells can effectively migrate and release growth factors to the damaged sites. Thus, positional stem cell transplantation therapy may prove more fruitful in this regard (Flax et al., 1998).

Many published studies report the successful use of stem cell transplantation strategies that have helped in the management of AD. One study showed that ESCs derived neural stem cells (NSCs) when transplanted into the mouse model of AD served as a better tool for the treatment for AD in comparison to using ESCs alone. The transplanted NSCs were stable, successfully differentiated into the cholinergic neurons and memory was found to be increased. On the other hand the vehicle group which received ESCs alone developed teratomas (Wang et al., 2006). Another study was carried out on the mouse brain that expressed aggregates of plaques and tangles. Genetically modified NSCs for expression of BDNF were successfully transplanted into the rodent brain which led to improved learning and memory. These NSCs did not decrease the level of the amyloid plaques instead the increased levels of BDNF helped in the formation a number of new synapses (Blurton-Jones et al., 2009).

Stem cells and iPSCs have been extensively employed to study the human specific responses and unravel the complexities of AD. In 2011, Yagi et al. first derived neurons from patient iPSCs which carried mutant (PSEN2) and (PSEN1) (Yagi et al., 2011). Since then a number of studies have been dedicated toward this approach of patient specific iPSC derived AD modeling. The catalytic subunit of the enzyme gamma-secretase is encoded by the gene (PSEN1) and mutated form of this gene results in the manifestation of the early stages of FAD (familial Alzheimer's disease). Stem cells models of the disease have largely targeted the involvement of gamma-secretase activity in the elucidation of sporadic as well as familial AD. For example, studies report that the Aβ42/40 ratios were higher in neurons derived from patient iPSCs (derived from PSEN1 mutant fibroblasts) in comparison to the healthy controls (Livesey, 2014). Similarly mutations in APP have also led to a similar increase in the Aβ42/40 ratios in the neurons of the human forebrain. A couple of studies have modeled FAD using iPSCs carrying mutations in the APP, such as dominant V717L and recessive E963Δ bearing mutation (Kondo et al., 2013) and the V717I mutation bearing iPSCs (Muratore et al., 2014). A study carried by Israel et al. demonstrated that inhibition of gamma or β-secretase activity led to a decreased production of Aβ40, however γ-secretase inhibition did not prevent phosporylation of tau proteins (Israel et al., 2012).

Stem cell derived neurons and astrocytes have been widely used to model FAD *in-vitro*. Gene mutations bring about observable changes in the cellular phenotype such as the Aβ peptide changes. However, how APP processing is interlinked with tau phosphorylation is an aspect which has not been efficiently modeled so far. The onset and initiation of AD is largely attributed to the amyloid hypothesis. APP is a single pass transmembrane protein and its proteolytic cleavage forms short Aβ peptides (Livesey, 2014). Mutation in the genes that are involved in the proteolysis of APP play a pivotal role in FAD. Aβ42 is the longer form of amino acid and its accumulation brings about neurodegeneration and cell death (Sproul et al., 2014). Post translational and cellular localization changes in the microtubule-associated tau protein play the second biggest role in AD progression. Tau changes and amyloid plaques if modeled together via stem cells will provide the best models of AD (Moore et al., 2015). Simple monogenic iPSCs' derived neurons are not sufficient to model amyloidosis since they accumulate low levels of toxic β-amyloid plaques. To overcome this hurdle stem cell lines bearing multiple mutations have been generated, which also over express the mutated genes such as APP and PSEN1.

## Parkinson's disease (PD)

Parkinson's disease ranks second after AD in being the most common and widely prevalent neurodegenerative disorder inflicting almost one percent of the aging population globally. Dopaminergic neuron loss from the nigrostriatum and substantia nigra pars compacta brain regions is the major characteristic of the disease (Marchetto et al., 2010). The other major hallmark being the presence of lewy bodies (α-synuclein aggregates) (Spillantini et al., 1998) which further promotes neuronal death due to the altered firing pattern of the neurons (Janezic et al., 2013). The genetic involvement of ubiquitin carboxy terminal hydrolase L1, serine threonine kinase 1, parkin, DJ-1, α-synuclein, and leucine-rich repeat kinase 2 have been reported in the case of genetically acquired familial PD (Dauer and Przedborski, 2003). Environmental influence in conjunction with age, genetic polymorphisms and chemical exposure predispose an individual toward sporadic PD, however the complex etiology is yet to be fully understood (Adami et al., 2014). Fitzmaurice et al. showed that variation in Aldehyde dehydrogenase enhances the pesticide effect related to PD thereby proving that environmental influences work in conjunction with genetics (Fitzmaurice et al., 2014).

Rigidity, resting tremor and bradykinesia are the major symptoms which make PD the most common movement disorder of the world affecting individuals post 65 years of age (Fu et al., 2015). Mechanistic and pathophysiological studies have given us a deep understanding of the disease. PD is generally associated with a disrupted calcium homeostasis, inflammation, disrupted kinase pathways, generation of reactive oxygen species, and dysfunctional mitochondria (Schapira et al., 2014; Xiao et al., 2016). Animal and cellular models have given us a deep understanding of the disease but data generated is not fully applicable to human subjects due to difference in disease pathogenesis of animals and humans (Devine et al., 2011).

PD so far has been managed using monoamine oxidase inhibitors, dopamine agonists, levodopa, and deep brain stimulation (Politis and Lindvall, 2012). The latter employs stimulation of the ventral intermediate nucleus, a part of the thalamus which can greatly reduce the symptoms of tremor. Other symptoms like rigidity and bradykinesia are also alleviated after the stimulation of the subthalamic nucleus or internal segment of globus pallldius. However, these treatments fail to repair the damaged brain region and the oral drugs are not effective beyond 5 years. Administration of L-DOPA (L-dihydroxy-phenyl alanine) can induce dyskinesis and fails to halt the disease progression (Politis and Lindvall, 2012).

The earliest transplantation studies employed the use of fetal ventral mesencephalic tissue of human origin (hfVM) which were engrafted in the striatum of the PD patients and laid the basis for cell therapy for PD. The attempts were successful and symptomatic relief was provided for almost 16 years in the successful cases. However, the clinical trials produced anomalies and the success of the approach was further challenged by the presence of side effects such as GIDs (graft induced dyskinesis). The reason was the presence of the serotonergic neuroblasts in the hfVM that led to an imbalanced serotonin/DA transporter ratios and false DA release (Politis and Lindvall, 2012). Studies also indicated that the survival of the transplanted fetal mesenchymal cells was very low and ethical issues further blurred the scope of this therapy. Since PD is characterized by a regional loss of dopaminergic neurons, transplantation, and replenishment therapy employing stem cell and iPSC derived dopaminergic neurons (yielding a pure population) in the SN region provides an excellent alternative.

Neurons with the DA phenotype have been developed from ESCs by using sonic hedgehog (Shh) and fibroblast growth factor-8 (FGF8) or the over expression of Nurr1 by using genetically modified NSCs (Kim et al., 2003). Co-culture of mouse bone marrow stromal and monkey ESCs have successfully yielded dopaminergic neurons in the past (Takagi et al., 2005). Studies further demonstrate the successful intrastriatal transplantation of fetal brain human NSCs in the MPTP lesioned monkeys which brought about improved behavioral changes (Redmond et al., 2007). A study reported that when NSCs were isolated from the patient brain, converted into dopaminergic neurons and re-implanted into the patient brain, the symptoms of trembling and rigidity were substantially reduced. The brain scans revealed an increase in the dopamine production by almost 58% and even when the levels did not increase further the symptoms did not revert back, hinting at a possible restorative potential of stem cell derived dopaminergic neurons (Hassan et al., 2010).

Transition from pluripotent stem cells to iPSCs has shown promising results and has opened new avenues for the modeling of PD. Dopaminergic neurons derived from patient specific iPSCs were successfully transplanted into a Parkinsonian rat striatum and showed a considerable reduction in motor asymmetry (Hargus et al., 2010). The role of mitophagy has been strongly implicated in PD. The mitochondrion targeted kinase PINK1 accumulates on the outer membrane of mitochondria on depolarization and further recruits Parkin which is instrumental in initiating mitophagy (Van Laar et al., 2010; Cai et al., 2012). The data generated on animal models so far has generated conflicts regarding the direct involvement of mitophagy in PD. However, PINK1 mutated dopaminergic neurons from human patient derived iPSCs have given us a clearer understanding of the mitophagy theory, thus proving once again that stem cell derived human disease models are far superior and far more edifying than any animal model (Seibler et al., 2011). It is also implicated that mitophagy may be a result of aging and may not have a direct correlation with the disease; however the hypothesis is still under contradiction. Progerin expression and long term culture have been employed to produce artificial aging of neurons in culture. These aged neurons have been treated as a model to study the late onset of PD and to elucidate the disease phenotypes (Miller et al., 2013).

As mentioned earlier, apart from disease modeling, stem cells, and iPSCs hold great promise as the *in-vitro* screening tools for therapeutic agents, drugs, and compounds. It has been reported that rapamycin, GW5074 (LRRK2 kinase inhibitor) and coenzyme Q10 diminish the cytotoxic effect of concanamycin A also known as valinomycin in patient derived iPSC neurons. It has been clearly seen that GW5074 does not reduce oxidative stress in the healthy neurons from control subjects whereas effectively lowers oxidative stress in the patient derived neurons bearing mutated PINK1 (Cooper et al., 2012). This difference underlines the importance of therapeutic compound screening in disease mutation bearing cells as well as the healthy cells. Another example where stem cells have been employed to study the PD physiology is the mutation correction of A53T α-synuclein via genome editing which diminished the formation of lewy bodies in iPSC derived dopaminergic neurons (Ryan et al., 2013). Mutation bearing iPSCs will thus serve as an excellent tool for screening and assessing the biosafety of drugs and compounds as well as identifying the underlying signaling cascades and novel therapeutic targets. The generation and characterization of iPSCs is cumbersome and the differentiated population of dopaminergic neurons may contain traces of the undifferentiated cells which may lead to teratoma formation. Thus, if patient derived fibroblasts are directly converted to dopaminergic neurons, the limitations with iPSCs can be overcome (Han et al., 2015). The successful differentiation of fibroblasts into the DA neurons are reported in literature and has paved way for potential disease modeling (Caiazzo et al., 2011; Kim et al., 2014). The stem cell technology can be used to identify the biochemical markers of the disease and can thus help in the diagnosis of early PD (Xiao et al., 2016).

## Amyotrophic lateral sclerosis (ALS)

A fatal neurodegenerative disease ALS is caused due to the motor neuron degeneration in the spinal cord, brain stem, and the primary motor cortex (Thomsen et al., 2014) which results in muscle wasting, paralysis and eventually death due to respiratory failure (Hedges et al., 2016). First described by Charcot in 1874, ALS is also known as Lou Gehrig's disease (Marchetto et al., 2010). ALS has been linked with FTD (frontotemporal lobar dementia) due to symptomatic, clinical, genetic, and pathological overlap. ALS and FTD when occur together further shorten the life span of a patient further. The two diseases are often considered the two ends of the same disease spectrum. Ling et al. gave evidence at the genetic level by reporting that FTD-ALS and ALS patients carry similar mutated genes (Lee and Huang, 2015). Many gene mutations are responsible for causing familial ALS such as mutations in PFN1, FUS/TLS (fused in sarcoma/translocation in liposarcoma), TARDBP or TDP-43 (TAR-DNA-binding protein 43), UBQLN2, C9ORF72, SOD1 (superoxide dismutase 1), HNRNPA1, OPTN, and VCP (Adami et al., 2014). Recently a new gene named TBK1 has been discovered which plays a crucial role in inflammation & autophagy which are inherently associated with ALS pathogenesis (Cirulli et al., 2015). Pathogenesis of sporadic ALS is attributed to glutamate excitotoxicity, protein mitochondrial dysfunction, aggregation, oxidative stress, deficiency of neurotrophic factors, glial cell dysfunction, and impaired axonal transport, all of these together eventually lead to the accumulation of intracellular neurofilaments (Kiernan et al., 2011; Robberecht and Philips, 2013). Riluzole is the only therapeutic drug commercially available which helps in the disease management, but its effect does not last beyond 6 months (Cetin et al., 2015). ALS has been widely studied using animal models; however reported failure of clinical trials has somehow restricted the sole dependency on *in-vivo* research (Gordon and Meininger, 2011). Patient specific iPSC banks hold promise for personalized medicine and are a good alternative for screening the efficacy of a number of drugs and compounds for the treatment of ALS (Giri and Bader, 2015).

Transplantation therapy employing stem cells can be effectively used as a therapeutic measure to deal with the devastating disease. Mesenchymal stem cells and hematopoietic stem cells have been efficiently employed as transplants in the affected spinal cord and have favorably supported ALS management (Mazzini et al., 2012). However, studies were conducted on a small group of patients and thus thorough research continues so as to be applicable for a larger pool of patients. Neural stem cells (NSCs) ESCs, glial-restricted progenitor cells (GRPs), and induced pluripotent stem cells (iPSCs) also offer a potential alternative for transplantation approaches and can be used (Traub et al., 2011). It is hypothesized that when donor cells are engrafted near the damaged motor neurons, they not only have an immunomodulatory effect but secrete trophic factors which improves the overall therapeutic potential of the transplant. Such transplants can effectively delay the progression and even the initiation of the disease (Teng et al., 2012).

The direct or peripheral injection of MSCs into the spinal cord of patients serves as a potent treatment for ALS and several studies have reported the therapeutic potential of MSCs (Mao et al., 2015). Several studies report the beneficial aspects of transplantable MSCs, which are used to deliver the required neurotrophic factors aiding in the prevention of motor neuron loss, improve survival of experimental animals, and delay the disease progression (Zhao et al., 2007; Vercelli et al., 2008; Uccelli et al., 2012; Krakora et al., 2013). Genetically manipulated MSCs which secrete GDNF (glial-derived neurotrophic factor) have been reported to increase the life of ALS rats by rescuing motor neuron loss (Suzuki et al., 2008). “NurOwn” developed by BrainStorm Cell Therapeutics are specialized MSCs which can secrete neurotrophic factors, can successfully differentiate into neuronal cells and can be used for ALS treatment. The cells are under the clinical trial phase (Therapeutics, 2015). A number of clinical trials have been conducted to assess the efficacy and safety of MSCs and many are still in process.

Neural stem cells have a specific lineage commitment for the cells of the CNS and find widespread applicability in the neurodegenerative disorders. When engrafted in the animal models of ALS, NSCs have been reported to exert a protective effect on the adjacent motor neurons (Hefferan et al., 2012). Stem cells not only provide symptomatic relief but help in restoration of the brain damage via repair and neurogenesis which is triggered in the affected spinal cord. Successful transplantation of neural stem cells from aborted fetuses into the patient's spinal cord has been reported (Xu et al., 2012). Results of the phase I clinical trials have been documented and show that stem cell therapy is a reasonably safe and can be used to treat a large enough pool of patients (Mazzini et al., 2015).

The familial cases of ALS can be modeled by ESCs harboring the disease mutations but the sporadic cases require the use of patient specific iPSCs. Literature reports the use of iPSCs for ALS modeling and hints at a possibility that motor neurons derived from patient specific iPSCs can be employed for the recapitulation of disease phenotypes. ALS exhibits a complex physiology and thus requires use of more than one cell type for its modeling. It was shown that SOD1 bearing astrocytes (from ESCs) exerted toxicity on the adjacent motor neurons (Wada et al., 2012) whereas iPSCs derived astrocyte bearing the TDP-43 mutation exerted no toxicity (Serio et al., 2013), hinting at the relevance of the co-culture of cells for modeling the complex disease. One drawback with *in-vitro* disease modeling of ALS is the short survival duration of motor neurons in culture which limits the study of phenotypic signs occurring in the aged diseased tissues. However, if iPSC derived motor neurons are grafted in the animal model it will increase the survival of these cells. The grafted cells can be recovered lately to visualize the disease phenotypes in the post mortem rodent tissues, thus offering a possible solution (Coatti et al., 2015).

Studies report that iPSC derived glial rich population of neural progenitors can be successfully transplanted into the spinal cord of mice suffering from ALS. These transplanted cells show a good survival, differentiation potential, and also enhanced the life span of the treated animals (Kondo et al., 2014). Stem cell therapy has been an area of debate for a long time. The beneficial aspects cannot be overlooked, but extensive clinical trials are in progress so as to generate an effective treatment and possible cure for ALS in the near future.

## Huntington's disease (HD)

Huntington's disease is a fatal genetic neurodegenerative disorder with no cure so far. Genetically acquired in an autosomal dominant manner, the disease is caused by an increased trinucleotide CAG (encoding polyglutamine) repeat in the ITI5 huntington gene. The CAG repeats are normally less than 36 in a healthy individual however a repeat of more than 40 will predispose an individual toward the disease. The striatum, cortex, substantia nigra pars compacta, substantia nigra pars reticulata, globus pallidus are the major brain regions which are subjected to severe degeneration (Carter and Chan, 2012). Enkephalin and gamma-aminobutyric acid containing medium spiny neurons, glutaminergic, GABAergic, and parvalbuminergic striatal projection neurons are severely affected. Mutated Huntington protein forms aggregates in the nuclei and cytoplasm of the brain tissues. Being a Cognitive, movement, psychiatric disorder the disease brings about mitochondrial, synaptic, and axonal transport dysfunction. The disease leads to a severe transcriptional dysregulation, proteolysis, and excitotoxicity (Peng and Zeng, 2011).

The disease, though incurable can be managed via gene therapy and drugs to alleviate symptoms. Drugs provide temporary symptomatic relief and largely target the motor aspects of HD. Tetrabenazine is dopamine depleting in nature and is used to reduce chorea, however severe side effects restricts its widespread use as a drug of choice for HD (Frank, 2014). The pros and cons of the various animal models of HD have been reviewed by Pouladi et al. In 2013 (Pouladi et al., 2013). HD has been widely modeled via rodent models *in-vivo*, however here too the success of the clinical trials has been limited. The problem lies not only with the animal model chosen but also with the robustness of the preclinical studies (Menalled and Brunner, 2014).

Gene therapy employs RNAi mediated silencing and reduction in the mutated HTT protein translation. However, gene therapy is effective only at the earliest stage of the disease but the symptoms are not visible until the later stages by which the major damage is already done. Replenishing the stratial neuron population by intracranial transplantation is an alternative cell therapy approach. Fetal striatal tissue grafts have been employed in the non human primate and rodent HD models as a means of transplantation therapy (Chen et al., 2014). These grafted tissues have a low survival rate and since they are procured from aborted fetuses, ethical constraints are high. Limited availability of donors further restricts the scope of this promising strategy. Neural progenitor cells (NPCs) derived from stem cells and iPSCs are a good alternative in this regard.

Successful differentiation of human ESCs into the GABAergic and DARPP32 positive medium spiny neurons (MSNs) is well reported. NPCs transplanted into the chemical lesion mouse model of HD showed efficient connectivity with the host neurons and also alleviated motor deficits (Ma et al., 2012). Another study by Carri et al. reports the successful differentiation of ESCs and iPSCs into MSNs carrying adenosine and dopamine receptors (Carri et al., 2013). However, NPCs transplantation has been more successful in chemical lesion HD mouse models as compared to the transgenic (R6/2) mouse model, the reason speculated being the short life span of the transgenic mouse (El-Akabawy et al., 2012). Along with disease modeling and regenerative transplantation therapy, stem cells and iPSCs can be simply used as an *in-vitro* tool for screening the efficacy and biosafety of drugs and small molecule compounds that help in relieving the symptoms of the disease as well as bring about a reduction in neurodegeneration (Carter and Chan, 2012). HD patient specific cell lines have been generated which recapitulate the disease specific phenotypes more closely (Consortium, 2012).

The gene therapy for HD involves targeting the mutant HTT allele (mHTT) transcript by RNA interference and ASOs (antisense oligodeoxynucleotides) specifically without disturbing the normal HTT allele transcript (Carroll et al., 2011; Hu et al., 2012). The exact role HTT in normal conditions is still not clearly understood but a study reports that HTT knockout mice do not survive and are lethal embryonically. Thus targeting only the mutant allele is preferable. ASOs target complimentary mRNA via RNase H mediated degradation and are important in research as they are capable of targeting the SNPs (single nucleotide polymorphisms). These SNPs are responsible for the difference between normal and mutated HTT gene alleles (Carroll et al., 2011). Studies have been carried out in the non human primate and rodent models of HD and disease alleviation was observed (Kordasiewicz et al., 2012). This paves way for a possibility that patient derived iPSCs carrying the disease mutations can be effectively corrected via ASOs and gene silencing and the NPCs thus derived can be successfully transplanted. Thus stem cell therapy in amalgamation with gene therapy can help in the management of the incurable disease in future (Chen et al., 2014).

## Three dimensional (3D) stem cell based studies: application in neurodegeneration and neurodegenerative disorders

*In-vitro* stem cell based studies are the best possible alternative to animal models because cells of human origin are used and thus data generated can be easily extrapolated to human subjects. However, two dimensional *in-vitro* studies have many limitations. The 2D culture comprise a homogenous population of cells, complex intercellular interactions are lacking, the cultures do not represent the complex microenvironment of an organ like the brain and the intact organ physiology is lacking. In this regard 3D cultures have played a pivotal role in overcoming the hurdles of typical 2D studies and also find widespread application in the field of neuroscience. Advances have been made to recapitulate the brain development and literature supports successful formation of stem cell derived 3D cortical and cerebral organoids (Lancaster et al., 2013; Sasai, 2013; Lancaster and Knoblich, 2014).

AD has been extensively modeled using stem cell and iPSC derived 2D neuronal cell culture however, a major limitation being the diffusion of Aβ aggregates, which are gradually, washed off with successive media changes. Therefore, it is hypothesized that 3D stem cell models of AD will provide a more compact and comprehensive brain microenvironment where the local niche will allow a sufficient Aβ aggregation (D'avanzo et al., 2015). This theory has been confirmed lately, where 3D matrigel system has been used for the culture of ReN cells to model familial AD. The 3D stem cell model of AD showed a significant deposit of β-amyloid plaques in a 6 week differentiated culture. The cells further showed elevated levels of phosphorylated tau protein thereby confirming that 3D based stem cell models are more efficient in recapitulating the disease pathophysiology (Choi et al., 2014). A study by Zhang et al. reported the successful recapitulation of *in-vivo* like responses in a 3D culture model of AD. The study comprises neuroepithelial stem cell derived neuronal culture in a PuraMatrix hydrogel comprising self assembling peptide matrix and laminin. The study confirmed that 3D microenvironment allowed Aβ sensing via p21-activated kinase (Zhang et al., 2014). Genetically engineered hNPCs production and 3D cell culture protocols for AD modeling have been standardized and published (Kim et al., 2015).

**Figure 2:**
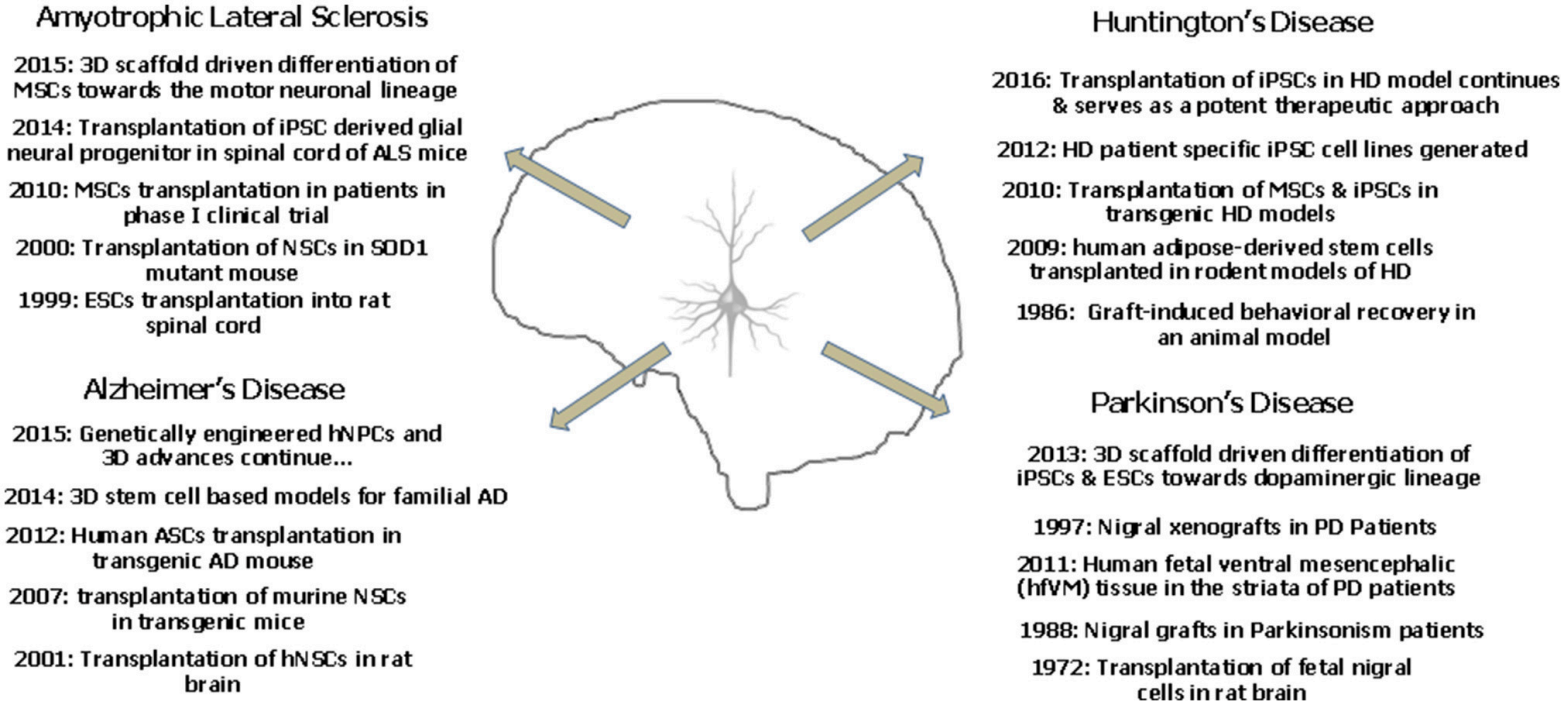
**The landmark studies/discoveries (5 each) related to the major neurodegenerative disorders and the possible role of stem cells in a time oriented manner**.

It is well reported that mouse iPSCs and ESCs show a better dopaminergic differentiation potential in a three dimensional peptide derived nanofibre scaffold. The 3D culture provides a better environment for the development of the DA neurons which showed appropriate action potential firing and expressed the specific markers as well (Ni et al., 2013). Successful differentiation of chorion derived MSCs into motor neurons has been recently reported in 3D nanofibrous gelatin scaffold. These motor neurons shall provide a possibility to model ALS in a three dimensional scenario (Faghihi et al., 2016). Figure 2 depicts the landmark studies/discoveries (5 each) related to the major neurodegenerative disorders and the possible role of stem cells in a time oriented manner.

## Concluding remarks

**Figure 3:**
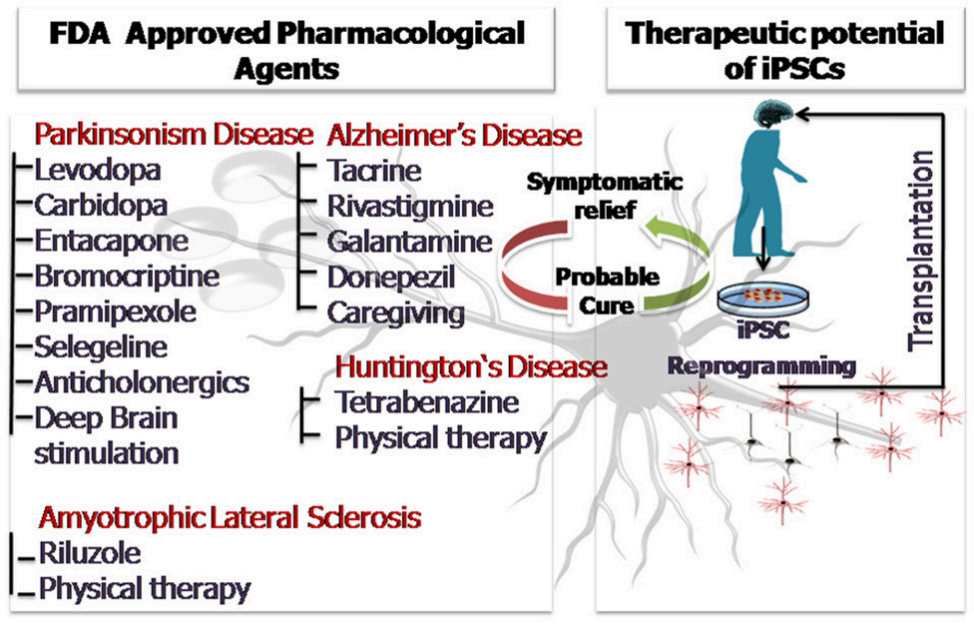
**The diagrammatic illustration of possible therapeutic strategies against the prevalent neurodegenerative disorders (ND)**. The nature of ND is progressive in nature and limits the clinical utility of pharmacological drugs which provide only symptomatic relief. Stem cell therapy and iPSC technology could harness neurorestorative and neuro regenerative relief for the patients suffering from neurodegenerative disorders as well as pave way for a possible cure in future.

The above discussion so far clearly sheds light on the widespread applicability of stem cells and induced pluripotent stem cells in the field of neurodegeneration. Disease modeling, transplantation therapy, restoration of lost brain functionality due to injury and aging and regenerative therapeutics are some of the areas where stem cells have been abundantly used. The article highlights the advances made especially in the past 5 years as envisaging the entire applicability of stem cells in neurodegenerative medicine is beyond the scope of the present discussion. Human stem cells and patient derived iPSCs have been instrumental in overcoming the major limitations of animal based research providing a more profound understanding of the neurodegenerative disorders. Patient derived iPSCs are even better models for understanding the disease pathophysiology and mechanistics because they carry the patient's genotype, bear the disease mutations and also account for the environmental influences. Stem cells have also been employed as simplistic *in-vitro* tools for screening of therapeutics and drugs. Three dimensional stem cell based studies and stem cell derived organoids have further contributed by providing a more *in-vivo* like microenvironment which is the closest possible mimic of a live animal. With technological advancements and efficient imaging techniques have revolutionized the concept of 3D stem cell based organoid research. Pharmacological intervention utilizing natural agents like curcumin has shown neuroprotective efficacy in clinical and experimental models of neurotoxicity and can provide beneficial effects in the neurodegenerative disorders in future (Srivastava et al., 2014). However, the current prevalent pharmacological treatments provide symptomatic relief only for a limited period of time and the drugs administered may have side effects. The advent of stem cell therapy has laid the foundation keystone for a possible cure with minimized side effects. Personalized medical treatment using iPSCs is the current face of modern medicine and constant efforts are being made to scale down the cost and increase the efficacy of the approach. Animal based clinical field trials cannot be completely surpassed and transplantation therapies will require validation. However, if cells of human origin are employed for the preliminary disease modeling and therapeutic screening, a lot shall be saved in terms of funds, resource, time and even animal lives. It would not be an exaggeration to say that with the proficient and judicious use of stem cells and iPSCs lesser animals shall be sacrificed and the rate of clinical trial failure shall be curbed. This shall lessen the moral as well as the economic burden. We still hope that future research will come up with effective cures for the fatal neurodegenerative disorders, until then efficient and affordable disease management and treatment can ensure a longer and healthier life for the aging population. Figure 3 is a diagrammatic illustration of possible therapeutic strategies against the prevalent neurodegenerative disorders (ND). The nature of ND is progressive in nature and limits the clinical utility of pharmacological drugs which provide only symptomatic relief. Stem cell therapy and iPSC technology could harness neurorestorative and neuro regenerative relief for the patients suffering from neurodegenerative disorders as well as pave way for a possible cure in future.

## Author contributions

SS drafted and prepared the manuscript, AS reviewed the draft, PS, YD, and CR prepared graphics. AP and DK helped in compilation of literature. All the authors have discussed the manuscript and agree to be accountable for the content of the work.

### Conflict of interest statement

The authors declare that the research was conducted in the absence of any commercial or financial relationships that could be construed as a potential conflict of interest.
